# Shifting students toward testing: impact of instruction and context on self-regulated learning

**DOI:** 10.1186/s41235-023-00470-5

**Published:** 2023-02-13

**Authors:** Patricia M. Simone, Lisa C. Whitfield, Matthew C. Bell, Pooja Kher, Taylor Tamashiro

**Affiliations:** Santa Clara University, Santa Clara, CA 95053 USA

**Keywords:** Self-regulated learning, Instructions, Online learning

## Abstract

Much of the learning that college students engage in today occurs in unsupervised settings, making effective self-regulated learning techniques of particular importance. We examined the impact of task difficulty and supervision on whether participants would follow written instructions to use repeated testing over restudying. In Study 1, we found that when supervised, instructions to test resulted in changes in the self-regulated learning behaviors such that participants tested more often than they studied, relative to participants who were unsupervised during learning. This was true regardless of the task difficulty. In Study 2, we showed that failure to shift study strategies in unsupervised learning was likely due to participants avoidance of testing rather than failure to read the instructions at all. Participants who tested more frequently remembered more words later regardless of supervision or whether or not they received instructions to test, replicating the well-established testing effect (e.g., Dunlosky et al. in Psychol Sci Public Interest 14:4–58, 2013. http://doi.org/10.1177/1529100612453266). In sum, there was a benefit to testing, but instructing participants to test only increased their choice to test when they were supervised. We conclude that supervision has an impact on whether participants follow instructions to test.

Improving memory performance is a goal for many people, particularly those in academic settings. Fortunately, scientists in many fields (e.g., cognitive, education, behavioral) have identified best practices to maximize learning and remembering. Dunlosky et al. (2013) reviewed the efficacy of ten easy-to-use learning techniques, derived from basic research in cognitive psychology, for their potential to help students achieve their learning goals. Based on a review of published studies, Dunlosky et al. (2013) found that some study tools reportedly used by many have limited utility (e.g., highlighting, summarizing, mnemonics, and re-reading). Of ten successful techniques identified, repeated testing was one practice found to benefit all types of learners of different ages and abilities. Despite the fact that students may not naturally recognize the power of repeated testing (Karpicke et al., 2009; Tullis & Maddox, 2020), getting students to use this technique could be as simple as instructing them to do so.

Ariel and Karpicke (2018) examined whether students would engage in repeated testing if they were informed of the utility of this learning strategy prior to studying. College-aged students were given instructions regarding the benefits of using multiple retrievals (three) to improve recall performance. Their performance was compared to students in a control condition who did not receive explicit instruction about study strategies. All students were tasked with learning 20 Lithuanian-English translations and allowed to choose their study strategy for each word pair. Participants were able to choose how to study the word pairs (either see both words together or be cued with the Lithuanian word and respond with the English translation). They found that participants who received instruction about the benefits of repeated testing tested themselves more often and remembered more of the translations during the cued recall task 15 min later. In a second experiment, they demonstrated that participants who were instructed to use testing in an initial task continued to use it in a similar task (learning Swahili–English word pairs) a week later without prompting, leading the authors to conclude that instruction to test has a lasting impact.

One aspect of student learning that has changed significantly since Ariel and Karpicke published their findings in 2018 is the need for students to regulate their own learning in fully online environments and without instructor supervision. As the COVID-19 pandemic necessitated significant changes to online learning, we wondered whether the instructions participants were given about the value of repeated testing would work as effectively in that context to produce the same increases in testing behavior Ariel and Karpicke (2018) saw in their lab-based intervention.

Likely to last well beyond the pandemic, online testing may be particularly useful in helping students gain extra practice with material outside of class so that they are more prepared to participate in class discussions that require higher level thinking. Even prior to the pandemic, a number of researchers found improvements in student learning using online practice tests that could lead to higher grades in a real course (Gurung, 2015; McDaniel et al., 2012; Van Camp & Baugh, 2014; but see also Bell et al., 2015, 2016). For example, Gurung examined online packages from three different publishers and found positive correlations between time spent using these online tools and students’ performance on in-class assessments, even when controlling for GPA.

Van Camp and Baugh (2014) examined the use of publisher-provided online learning tools (*MyPsychLab*, Pearson) in their Introductory Psychology course. Students not only reported that they believed the tools helped them to learn, they also reported that they enjoyed using the online tools as part of the course. While they found that students who chose to use the tools received better grades than students who had not, there was no improvement in the overall course grade or passing rate when the tools were *required* for the class, and importantly, not everyone used the course tools when they were available or required (Van Camp & Baugh, 2014). One possible conclusion is that some students, perhaps those who are already more skilled in learning strategies and/or more motivated to do well, use tools when they are available, whereas less skilled or motivated students do not. We note that these publisher-provided online study tools include more features than merely quizzing, although quizzing is an important component. It is possible, based on Ariel and Karpicke’s (2018) findings, however, that regardless of motivation to do well, if all students were explicitly informed about the benefits of testing right before using the quizzing tool, this might increase the use of testing among all students and thus, improve all student performance.

We explored this phenomenon further in our first study thereby testing the effectiveness of an instruction intervention on participants’ self-regulated learning across different contexts. We asked all participants to try hard to learn 20 English-Swahili word pairs across two modalities: some completed the tasks under supervision in the laboratory (thus, replicating Ariel & Karpicke, 2018), and some worked on their own, unsupervised. As noted above, Ariel and Karpicke (2018) found that students selected repeated testing when they were provided with instructions regarding the superiority of this learning strategy just prior to engaging in an opportunity to learn word pairs in a laboratory setting and a week later in a similar laboratory setting. Whether students would follow this instruction prompt in an online, unsupervised setting in the same way they did under supervision in a laboratory setting is not yet known.

A second feature that we explored was whether the difficulty of the task would change students’ adherence to the instructions to use testing as a strategy to improve learning. In their first experiment, for example, Ariel and Karpicke (2018) cued participants with a Lithuanian word and then, asked them to recall and type in the English pair. Recalling English words given a non-English word as a cue is an easier task compared to recalling a word in an unfamiliar language given an English word as the cue (Bangert & Heydarian, 2017; Nelson & Dunlosky, 1994). It is important to know if task difficulty influences student decisions about testing and whether providing information about the value of testing at the start of a study session has any influence over behavior in that situation. Based on previous work cited above using publisher-provided online learning tools, we anticipate that not everyone will use the resources available to them, even with instruction. Other laboratory-based studies have shown that students *will* choose to test themselves but only after they have already reached a certain level of recall based on just viewing the word pairs (Kornell & Bjork, 2007), or if they are allowed to receive hints (Vaughn & Kornell, 2019) to make the learning task more “fun.” Therefore, we anticipate that participants will choose the study option more often in the difficult task (recalling Swahili) than in the easier task (recalling English words).

We examined the durability of the instruction effect, i.e., whether students will continue to select testing over studying when instructed to do so, and whether that effect is constant across different contexts (online vs. in person) and under differing levels of task difficulty (easy vs. hard). We examined the effects of not only task instruction about testing effects (present or not) but also the effects of task context (supervised or unsupervised) and task difficulty (easy or hard) on participants’ decisions to use testing as a strategy to learn word pairs. In each context and difficulty level, participants were randomly assigned to one of two groups. Either they received instruction about the benefit of testing, suggesting that testing themselves was the most effective strategy to learn the words (instruction group), or that students should learn the word pairs, so they could recall as many as possible later (control group). We manipulated task context by having students complete the study with supervision in a laboratory or on their own and unsupervised. We manipulated task difficulty by cuing recall with the Swahili word and asking for the English translation (easy task) or cuing recall with the English word and having participants recall the Swahili translation (hard task).

## Study 1

### Method

#### Participants and design

We used G*Power to determine the appropriate cell size based on effect sizes reported by Ariel and Karpicke (2018). This yielded a target of 30 participants in each condition. The majority of students identified as female (69%) and were mainly first and second year students (84%). See Table 1 for specifics for each condition. Participants were undergraduate students in introductory psychology courses who completed the study for course credit.

**Table 1:** Number of participants and demographics for each condition

Context	Difficulty	Participants		Gender (% Female)	Percentage first yrs
		Control	Instruction		
Supervised	Easy	30	31	70.5	54.1
	Hard	38	38	72.4	50.0
Unsupervised	Easy	30	29	61.0	61.0
	Hard	32	30	71.0	43.5

The Easy condition consisted of Swahili prompts with participants reporting the appropriate English word, while the Hard condition had English prompts for the appropriate Swahili word. The Instruction condition explicitly emphasized the benefit of testing (whereas the Control condition did not). Study conditions were conducted one at a time, and participants were randomly assigned to instruction versus control. Although random assignment was not possible for the context and difficulty manipulations, participants were not able to self-select which condition to join, decreasing the likelihood of selection bias. Participation in one condition excluded them from participating in another.

#### Materials

All procedures used 20 Swahili–English translations (e.g., *jabini-cheese*), all nouns. The Swahili words were six letters and were of medium difficulty for recalling English when prompted with Swahili according to previous research (Bangert & Heydarian, 2017). Task difficulty varied according to the language of the word to be retrieved following the cue, English (easy) or Swahili (hard). Context was either supervised in a laboratory setting or unsupervised on their own.

The program was created using PsychoPy (Pierce et al., 2019) for in person testing and was converted to an online version using Pavlovia as the platform for unsupervised learning. For all participants, we collected demographic information via an online questionnaire.

#### Procedure

For participants in all conditions, the first screen told them they would be learning 20 Swahili–English translations. They were told that they could control how they studied the translations in the learning phase and that their goal should be to learn all of the translations so that they would recall as many as possible on the final test that would follow approximately 45 min after the start of the experiment.

Before beginning the choice block, each participant was then randomly assigned the instruction or control condition. Participants in the instruction condition saw a second screen that included specific information about the benefits of repeated retrieval, recommending that they continue to practice the translations until they remembered each of them three times, modeled after the methods of Ariel and Karpicke as shown in their Appendix (2018, p. 56). The instructions were thus:

Before you begin, we wanted to tell you about a strategy that is extremely effective for learning: repeatedly self-testing. Research shows that people learn more from repeated testing than from repeated studying. This is illustrated in the Figure to the right which shows differences in final memory performance for students who repeatedly studied information vs. repeatedly retrieved information with practice tests. The best strategy to ensure that you remember all the translations on the final test in 45 minutes is to successfully retrieve each translation at least 3 times across multiple practice tests. You should not stop studying a translation until you have remembered it at least 3 times.

The control condition received no second screen with this information and proceeded immediately to the first phase of the experiment.

**Fig. 1:**
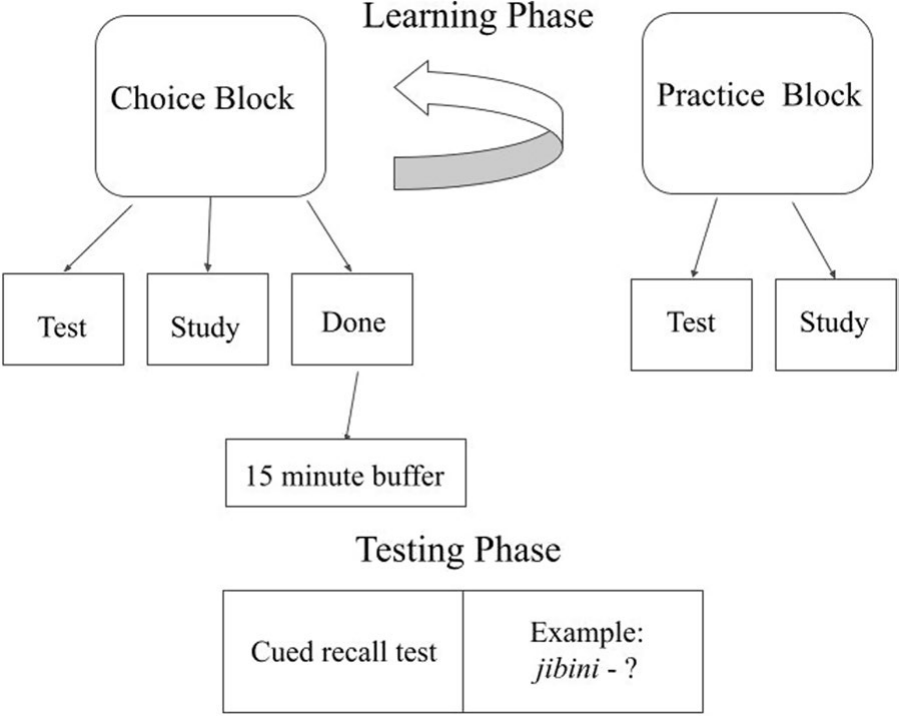
Procedure schematic

The study began with a learning phase which consisted of alternations between a choice block and a practice block (see Fig. 1). The choice block involved sorting the word pairs into Study, Test, and Done piles. Participants sorted by clicking on the blank pile to view the word pair, and then, they selected how or if they wanted to review that word pair by clicking on Study, Test, or Done on the right. Once all the word pairs were sorted into one of the three piles, the practice block began by asking if the participant would like to start with Study or Test piles first (if word pairs were placed in both piles, otherwise they proceeded directly to the pile that contained the word pairs they had selected for practice). Word pairs placed in the Study pile were presented in random order on screen until the participant pressed Enter to move to the next pair. Word pairs placed in the Test pile were randomly presented with only one word (Swahili or English, depending on whether they were in the hard or easy condition) shown and a blank area beneath it for the subject to type the appropriate translation. Once Enter was pressed, the participant was asked if they would like to receive feedback about their response. If Yes was selected, they were shown their answer and the correct answer for 2 s before the next trial began. Completion of the practice block brought the participant back to the choice block. This alternation between choice and practice blocks continued until the participant placed all word pairs in the Done pile during the choice block.

For supervised learning, participants then moved to a new computer in the same room to complete unrelated distractor tasks for 15 min. For unsupervised learning, they were directed to watch a YouTube video about visual illusions that lasted 15 min. After the distractor task, participants completed the testing phase of the study. Participants were cued with a Swahili word if they were in the easy condition and asked to type in the corresponding English word (e.g., jibini-?), whereas those in the hard condition saw the English word and were asked to type the corresponding Swahili word (e.g., cheese-?). They were allowed as much time as they needed to type the translation. Once they hit Enter, another cue appeared until all word pairs were presented. The order of presentation was random. This cued recall was identical to how the participants tested themselves during the learning phase if they placed the word pairs in the Test pile except that no feedback was given during this cued recall test. Following this test, participants completed a survey requesting information on demographics, their study habits, how hard they tried on this task, and their familiarity with Swahili. Finally, they were debriefed and thanked for their participation.

## Results and discussion

### Study strategy

We sought to determine whether the instruction effect shown by Ariel and Karpicke (2018), namely increasing testing by telling students about the benefits of this technique, would persist if students were presented with those instructions in an unsupervised online task, and regardless of how difficult the material was to learn. Because participants chose a mix of test and study trials to complete this task, and we were interested in studying self-regulation of learning within individual participants, we examined the proportion of trials in which participants tested relative to the total number of trials they completed to create a test–study ratio measure. This test–study ratio measure takes into account how each participant chose to blend testing and studying activities in the different settings (supervised, unsupervised) and task difficulty levels (easy, hard).

We analyzed how often students used testing with a 2 (instruction vs. no instruction) × 2 (supervised vs. unsupervised) × 2 (easy vs. hard) ANOVA. To create a single measure of the degree to which students favored testing versus restudying, we used the mean proportion of test trials divided by total trials (study plus test) as our main dependent measure (see Table 2). Regardless of the total effort each participant put forth to learn, i.e., quitting after 20 trials versus 200 trials, this allowed us to see whether their study strategy “blend” favored testing. Scores closer to 1 indicate greater adherence to the recommendation to test, regardless of persistence in the task in general. This analysis yielded main effects of instruction, *F*(1, 250) = 8.43, *p* = 0.004, *η*_p_^2^ = 0.033, and context, *F*(1, 250) = 7.89, *p* = 0.005, *η*_p_^2^ = 0.031, and a three-way interaction among instruction, context and difficulty, *F*(1, 250) = 5.00, *p* = 0.026, *η*_p_^2^ = 0.020.

**Table 2:** Proportion of test trials selected during training and final test recall accuracy

Context	Difficulty	Proportion of test trials		Recall accuracy proportion	
		Control	Instruction	Control	Instruction
Supervised	Easy	0.62 (0.17)	0.66 (0.17)	0.89 (0.18)	0.96 (0.07)
	Hard	0.57 (0.18)	0.68 (0.16)	0.58 (0.36)	0.72 (0.27)
Unsupervised	Easy	0.49 (0.20)	0.62 (0.17)	0.76 (0.22)	0.77 (0.28)
	Hard	0.58 (0.24)	0.58 (0.22)	0.61 (0.34)	0.75 (0.26)

The data shown represent the mean (standard deviation) for each condition.

Although we did not predict a specific three-way interaction, we did expect that performance in unsupervised settings might differ from the supervised setting used by Ariel and Karpicke (2018) and specifically that students might comply with instructions less when not monitored. Therefore, we examined the mean proportion of test trials used within each level of context separately to understand this result with a 2 (instruction) × 2 (difficulty) ANOVA looking only at supervised participants. This analysis showed a significant main effect of instruction, *F*(1,133) = 6.21, *p* = 0.014, *η*_p_^2^ = 0.045, but no significant effect of difficulty of the task, *F*(1,133) = 0.32, *p* = 0.574, *η*_p_^2^ = 0.002, nor a significant interaction, *F*(1, 133) = 2.12, *p* = 0.148, *η*_p_^2^ = 0.016. As seen in Table 2, when under supervision, participants favored testing over studying when given instructions to do so. In contrast, a 2 (instruction) × 2 (difficulty) ANOVA looking only at behavior of unsupervised participants revealed no significant effects. Neither instruction, *F*(1,117) = 2.89, *p* = 0.092, *η*_p_^2^ = 0.024, nor difficulty of the task, *F*(1, 117) = 0.52, *p* = 0.470, *η*_p_^2^ = 0.004, nor their interaction *F*(1,117) = 2.78, *p* = 0.098, *η*_p_^2^ = 0.023, yielded significant differences in how participants chose to study the word pairs.

In summary, we found that instruction to test resulted in favoring testing for supervised participants regardless of task difficulty. Neither instruction nor task difficulty changed the behavior of unsupervised participants. Simply stated, students adhered to the recommendation to test when completing the tasks under supervision. Contrary to our prediction, task difficulty did not influence the instruction to test in supervised or unsupervised conditions.

### Effort

We examined effort in two ways: total number of trials completed during the study and self-report after the study (see Table 3). We ran a 2 (instruction) × 2 (context) × 2 (difficulty) ANOVA on the number of trials completed and found main effects of context and difficulty. Supervised participants used significantly more trials to learn the words than unsupervised participants (6.24 vs. 4.74), *F*(1, 250) = 10.49, *p* = 0.001, *η*_p_^2^ = 0.04. Participants given the hard task (recall Swahili) completed an average of 6.67 trials, whereas participants asked to recall English completed an average of 4.31 trials, *F*(1, 250) = 25.89, *p* < 0.001, *η*_p_^2^ = 0.09. Interestingly, we found that instruction to test did not increase the total number of trials participants completed (control 5.22 vs. instruction to test 5.76), *F*(1, 250) = 1.38, *p* = 0.241, *η*_p_^2^ = 0.005. As we noted above, instruction did change the blend of their choice of how to engage in the study in that those with instruction to test did test more frequently and they also reduced the number of study trials. There were no significant interaction effects.

**Table 3:** Mean total trials completed and self-reported effort

Context	Difficulty	Mean (SD) Total trials		Mean (SD) self-reported effort	
		Control	Instruction	Control	Instruction
Supervised	Easy	4.7 (2.7)	5.7 (3.3)	4.2 (0.75)	4.3 (0.86)
	Hard	6.6 (4.0)	7.9 (4.7)	4.1 (0.88)	4.3 (0.78)
Unsupervised	Easy	3.1 (1.8)	3.8 (1.9)	3.9 (0.78)	3.9 (0.74)
	Hard	6.5 (5.7)	5.7 (3.4)	4.1 (0.89)	4.1 (0.73)

The data shown represent the mean (standard deviation) for each condition. Total trials are study plus test trials. Self-reported effort is on a 5-point scale (5 being most effort).

To examine whether participants’ self-reported effort varied across conditions, we conducted a 2 (instruction) × 2 (context) × 2 (difficulty) ANOVA on the level of effort participants reported putting forth when asked about this following the final recall task. The context in which participants completed the task (supervised vs. unsupervised) was a significant predictor of their reported effort. We found that, on a 5-point scale with 0 being the least (“no effort”) and 5 being the most (“I tried as hard as possible”), supervised participants reported trying harder than unsupervised participants (*M* = 4.23, *SD* = 0.82 and *M* = 3.99, *SD* = 0.79, respectively), *F*(1, 250) = 5.83, *p* = 0.016, *η*_p_^2^ = 0.022. There were no effects of instruction or difficulty on this measure.

### Recall accuracy

Although not a main focus of our study, we were also interested in whether those who favored testing over studying actually recalled more words. Because students in the control condition also engaged in testing, to verify that we replicated the testing effect more broadly we examined the correlation between the proportion of times participants chose to test themselves and proportion correct at final recall regardless of where the learning took place (supervised or unsupervised), which was significant, *r* (256) = 0.404, *p* < 0.001. Although as we noted above that instruction did not change behavior in the unsupervised context, testing helped all participants remember more regardless of supervision. This finding is consistent with the well-established benefit of the testing effect and did not depend on where students completed the task. In other words, there is a benefit to testing, even unsupervised, but instructing participants to test will increase their choice to test only when supervised.

## Study 2

The methodology of Study 1 precludes us from determining whether participants were ignoring the instructions to test or if they were not reading the instructions. It is possible, for example, that unsupervised participants did not read the instructions at all, or they did read them but chose not to follow them. We designed a second study with the simple goal of determining whether participants read instructions in supervised vs. unsupervised contexts. To achieve this, our goal was to have the instructions make the task much easier (by giving them the answers within the instructions), so there would be a strong motivation to follow them if they were read.

### Method

#### Participants

Participants (*n* = 114) were undergraduate students in introductory psychology courses who completed the study for course credit. As with Experiment 1, the majority of participants identified as female (77%) and were mainly first and second year students (86%). Participants completed the task either in a supervised (lab) or unsupervised location (at home, the library, etc.). In the control condition, 28 participants were supervised and 30 were unsupervised. Experimental condition participants were also supervised (*n* = 29) or unsupervised (*n* = 27).

#### Materials and procedure

We intended this study to be perceived by participants as challenging, so we presented 40 English-Swahili translations to participants via a Qualtrics survey and told them they would be asked to enter the Swahili translation for the English words. All participants were initially told to report to a laboratory for testing, but half the participants, chosen at random, were informed in an email that the session was over-booked and they were to complete the task on their own (at home, library, etc.). Other participants were given the task in the laboratory where the experimenter was nearby to supervise the session.

Participants in the control condition first saw a set of instructions telling them they would be attempting to learn 40 English–Swahili word pairs. The instructions asked them to do their best to learn the pairs so that they would be able to recall the Swahili word later and that they would be given the choice to practice before the final test. Next, the word pairs appeared on the screen for up to 3 min before participants were asked if they wanted to practice the translations. If they selected that option, 40 multiple choice questions followed in which the English word appeared and the participant could select its Swahili translation from four choices. Corrective feedback was given after each question. The participant was then asked if they would like to practice for a second round. Finally, a cued recall task ensued in which an English word was given and participants had to type in the correct Swahili translation.

Participants in the experimental (answer given) condition received the same sequence of tasks in the survey, but with key differences. First, the instructions told participants about the value of testing over restudying, using the same information and graphic that we used in Study 1. However, the last paragraph of the instruction screen read as follows:

Second, the real purpose of this study is to find out if you are reading the instructions. We’d actually like you to skip the practice session(s) and go directly to the final test. Instead of typing in the Swahili translation for the English word you’re given, just type in the word ‘bronco’, in all lower case letters as it appears here, for all of the translations.

Thus, for participants in the answer given condition, a correct response, if they were reading and following the instructions, would be to say “no” to each question about whether they wanted to practice the translations, skipping those blocks entirely, and to enter “bronco” for the responses to each of the cued recall questions at the end of the survey. All participants were thanked and debriefed once they had completed the survey.

## Results and discussion

We first examined whether those receiving instructions followed them. We found that no one in the control condition entered the word given (‘bronco’), and so we are confident that anyone in the answer given condition who entered that word on the final test knew to do so from reading the instructions. We found that 71.4% of participants (40 of 56) did indeed enter the answer provided to them (‘bronco’) at least once. We are confident, then, that over 70% of participants read and followed the instructions, and conversely, that nearly 30% of the participants did not read the instructions.

A second measure of reading the instructions was whether or not participants engaged in any practice tests; those in the answer given condition were told to skip the practice tests and those in control were encouraged to do so. Using a Welch’s two-sample t-test, we found a significant difference between the number of practice blocks completed in the two groups (out of 2 possible), with participants in the answer given condition completing fewer (*M* = 0.45, SD = 0.63) than those in the control condition (*M* = 0.97, SD = 0.46), *t* (100.19) = 5.02, *p* < 0.001, Cohen’s *d* = 0.94, as we expected.

Recall, however, our results suggest that 30% of the participants in the answer given condition did not read the instructions. If that’s the case, then we would expect them to have engaged in more practice than those who followed our directive to skip the practice trials entirely. This is what we found. Using a Welch’s two-sample *t*-test, we found that the 30% of participants in the answer given condition who did not follow the instructions completed significantly more practice blocks, (*M* = 0.94, SD = 0.77), than the 70% who answered ‘bronco,’ (*M* = 0.25, SD = 0.44), *t* (19.00) = 3.35, *p* = 0.003, Cohen’s *d* = 1.10. Furthermore, we would also expect that participants who received the control instructions, which encouraged practice, would practice more than the 30% of participants in the answer given condition who did not read the instructions, therefore missing out on the encouragement to test. This was also supported. The 30% of answer given participants who did not read the instructions at all completed as many practice blocks (*M* = 0.94, SD = 0.77) as the control group (*M* = 0.97, SD = 0.46), Welch’s *t* (18.00) = 0.14, *p* = 0.891, Cohen’s *d* = 0.04. We also analyzed the influence of supervision on rates of compliance in the answer given condition. We found that participants did follow the instructions (i.e., used ‘bronco’) more often when supervised (79%) than unsupervised (63%) although not significantly so, *X*^2^(1,* N* = 56) = 1.83, *p* = 0.176.

We feel confident in concluding that nearly 30% of participants did not read the instructions, and we can extrapolate that these same rates would apply to the percentage of participants who read the instructions in the control condition as well. Reading instructions and following instructions are not the same especially when there is a reason to avoid the instruction (e.g., they require the participant to do something perceived as hard or unpleasant). We demonstrate in this study, using instructions that make the task much easier, that while a large majority of participants do read the instructions, not all do.

## General discussion

### Context matters

Our primary interest in this investigation was to determine whether or not we could get students to use an effective study technique, repeatedly testing themselves to learn new material, not only in a supervised setting but also when studying on their own, unsupervised. In short, we found that when students were informed about how useful testing is as a study technique, they did increase their use of it, but only when they completed tasks in a laboratory under supervision. When we gave them the same instructions about using testing, but in an unsupervised setting, they used testing about the same amount as those who saw no such information. The results of our second study show that this is likely due to participants reading but not wanting to follow the instructions, as testing is effortful, relative to studying. This outcome replicates and extends Ariel and Karpicke’s (2018) finding that instructions to test can be useful in changing how students choose to learn, clarifying that there may be common situations in which such instruction will not be followed. Given the recent and dramatic increase in the need for instructors to create effective learning opportunities students can engage in on their own time, this is a useful, if disappointing, finding.

We also found that the context in which students completed the task mattered for the level of effort they put into it, regardless of what technique they picked to learn. For example, in Study 1, using the total number of trials that students completed to try to learn the word pairs as an objective measure of persistence, we found that students learning in the laboratory gave more effort than students learning on their own. This was true regardless of whether they had been given specific instructions to test.

Participants who completed the task in a supervised environment experienced a host of circumstances that likely differed in meaningful ways from those completing the task unsupervised. For example, when supervised, we asked all participants to turn off their phones and put them away. We also told them that we would be watching from another room, ensuring that they knew they were under supervision. We do not know at this point whether the mere presence of an authority figure, the academic setting itself, the lack of distractions, or even the effort invested to travel to a specific building at a specific time to participate were most influential or if each of these factors played some important role. We note, however, as shown by Study 2, that the majority of students followed the instructions regardless of supervision when the instructions made the task easier to complete.

In courses that are taken fully online without supervision, there may only be a few of these factors under an instructor’s control. Trying to keep students from being distracted by other applications open on their desktops, notifications from other devices, or roommates and family members coming in and out of the room is likely a losing proposition. We suggest that instructors may have to rely on features of the task itself to keep students engaged or at least willing to go back to the task at a later time (which could be useful in and of itself due to the benefits of spacing out study sessions over time, e.g., Dunlosky et al., 2013). We consider some possibilities later in this discussion.

### Students completing difficult tasks studied harder, but not smarter

We expected that telling students about the benefits of testing might be particularly useful for those who were given a more difficult task. Therefore, in Study 1, we asked participants in some conditions to recall words in an unfamiliar language (to our participants) such as Swahili. We surmised that if the task was difficult, they might respond to that challenge by using a tried and true method we had just informed them about (for those in the instruction condition). They did not. Students who had to recall Swahili words did not add any more testing to the mix than students recalling English words, even if they had just read about the value of testing. This suggests that even when testing would be especially useful, as in the difficult task, because testing itself is effortful, students are still reluctant to do it.

It is reasonable to think that an alternative outcome could have also occurred with the more difficult task. Students may have found test trials more aversive than study trials when trying to remember more difficult material due to greater failure rates during the learning phase. As shown by Vaughn and Kornell (2019), when participants are given only two options for learning, test or study, they typically favor studying by large margins. Vaughn and Kornell have shown that participants will choose testing, however, when there is an option to get a hint about the word’s identity, presumably because this makes the testing event more likely to result in a success for the learner. Here, our participants do not appear to have gravitated more toward the study option to avoid failure. Rather, they kept the same ratio of study and test trials, and added more trials overall.

Given that students completing the difficult task were not avoiding testing by gravitating toward more study trials, it is perhaps all the more impressive that, in general, they persisted through more learning trials overall than students completing the easier task. That is, independent of what students actually did with a word pair (study it or test it), they did *more* of it. This suggests that students recognized they did not know the material yet and that they should keep at the task.

### Students who tested more also remembered more

Regardless of instruction, context or task difficulty, Study 1 demonstrates that students who tested more remembered more and provides further evidence for the benefit to testing: Students who engage in more testing remember more information than those who do not test as frequently.

Giving students the choice to study or test, as we did in Study 1, seems to be an important component to the benefit of testing. For example, Tullis et al. (2018) found that testing was more effective than restudying overall, but forcing participants to take a test did not enhance learning if they did not choose to be tested. Similarly, Vaughn and Kornell also noted that “For one thing, what matters is how students choose to study, not what they think is best for learning” (Vaughn & Kornell, 2019, p. 2). Getting students to test more when they have the choice to study or test is trickier.

### What could make students choose to test more?

How do we get students to choose to test? We surmise there are two distinct paths to get students to choose to test more. One is to persuade them to change their beliefs about testing by giving them instructions about its benefits, or even personalized feedback about how they have performed with testing versus studying in a task. For example, Tullis et al. (2018) found that although students consistently judged that restudying would yield better recall than retesting even though, across four experiments, restudying did *not* yield better recall in their actual performance. However, students revised their beliefs about retesting following clear and specific feedback about how many restudied versus retested items they actually had remembered in previous testing sessions. So perhaps consistent feedback about performance on tested vs. studied material can help, but this requires a lot of input from instructors.

The second path does not require any metacognitive awareness of the value of testing, but relies instead on making the testing experience itself less aversive, perhaps even fun. This is the approach taken by Vaughn and Kornell (2019) in their study, conducted online using mTurk. They allowed participants to select an option to receive a hint (one or more letters) when they retrieved the answer. None of their participants elected the no hint option, meaning that when given the option to restudy, test, or receive a hint, most people choose the less aversive hint option. In Experiment 2, when hints were not available, participants greatly preferred restudy versus test trials (80–20%, respectively). So clearly, participants are motivated to test, but they also want to get the answer right. If participants can be motivated to test, i.e., using dynamic features of the publisher-provided testing tool itself, then the need to persuade individual students of the value of testing may be unnecessary. Given our finding that instructions about the value of testing are more likely to be followed in supervised contexts, this second path offers a more “hands-off” approach for instructors who wish to help students improve their learning.

What may be needed at this point is a more direct test of how students manage various factors in self-regulated learning situations. Specifically, one could manipulate how effortful testing is (e.g., with difficult words, with hints), the social constraints of the situation (e.g., while an instructor is watching vs. not), and metacognitive information (instructions to test are given or not). We expect that instruction to test may not be helpful outside of the social constraints to do well (e.g., when alone) and may not be necessary when the task is fun (e.g., hints are used) or when the task is simple and testing is not warranted for learning. But when the task requires effort (e.g., no hints are given, the words are unfamiliar), supervision will increase the likelihood that students will follow instructions to test. We note, for example, that Ariel and Karpicke tested groups of 4–12 participants at a time in a laboratory setting and they found a significant and large effect of instruction on the decision to test. We also found a significant effect of instruction on testing when students were tested individually in a supervised lab context, but not when they were unsupervised. If participants in our unsupervised setting had been given an option to receive hints during testing, however, we expect this would have increased their use of the technique.

### Limitations

One limitation to our study is that we do not really know what students were doing in the task, particularly those who were unsupervised but even those who were supervised. For example, we observed students covering the screen on study trials, effectively making these test trials. If this occurred often, it would underestimate the level of testing in which students engaged, weakening our confidence in the validity of this method for assessing study strategies. We might be able to assume that students did this to an equal degree across all conditions. They may have chosen to test in this manner because it might have been less aversive than typing in an answer during a test trial and having to wait to get feedback that it was wrong. This “covert” testing possibly may have caused us to underestimate how many testing trials students used. We note too that Vaughn and Kornell (2019) also reported anecdotally that students engaged in this behavior in some of their laboratory experiments.

Second, we cannot determine why instructions did not work for unsupervised learners. Study 2 suggests that the majority of students, regardless of supervision, read the instructions. Following instructions depended on whether or not the instructions made the task easier (answer given) or harder (use testing). However, since this is exactly the situation in which quizzing tools are often used by instructors in their classes–study outside of class, flipped classroom, etc., we can conclude that getting students to follow instructions in unsupervised conditions may not be sufficient to change behavior, unless the instructions make the task easier.

Third, we were unable to conduct Study 1 using true random assignment. Study 1 participants were randomly assigned to instruction vs. control conditions; however, random assignment was not possible for the context and difficulty manipulations. Participants were not able to self-select which condition to join, decreasing the likelihood of selection bias. But because the conditions were run over a period of time, we cannot rule out the possibility of cohort effects having an influence on the results.

Fourth, we did not assess actual classroom learning. It is possible that were students given guidance about the value of testing from a regular instructor and where recalling the information could yield a higher course grade, participants might have tested more often even when unsupervised. There is reason to doubt that this would be sufficient, however. As mentioned previously, Van Camp and Baugh (2014) asked students to use publisher-provided quizzing tools in their introductory psychology courses but found that requiring use of the tools did not increase their use. It is unclear whether more students would use these tools to test themselves if they were initially given information about the value of the testing effect *in class* and *just prior to* being asked to use the tool on their own.

### Conclusion

In conclusion, our primary finding is that participants are more likely to follow instructions to test and they report trying harder only while supervised during in-person sessions. Our evidence suggests that the vast majority of participants are actually reading the instructions, but that whether instructions are followed depends at least in part on how difficult the task is they are being asked to do. If there is a single parameter to increase testing in unsupervised settings, we did not find it.

### Significance

The ability to learn and remember content is a cornerstone of education, and improving this ability is important to students and their professors alike. There is ample evidence that students recall more information when they test themselves (e.g., quizzing), as opposed to re-reading or restudying the information. These studies provide evidence that instructors can increase the amount of testing students do, but only under certain conditions. For example, when we tried to increase the number of times students used testing by giving them instructions about its benefits at the start of a task, students did test more, but only when their learning was supervised. When learning on their own, students who saw these same instructions did not increase their testing; they kept the same “test-study ratio” as those who were given no information about the usefulness of testing. This pattern stayed the same regardless of whether the task was easy or difficult. In a second study, we found that most participants did read and follow the instructions we gave them when these instructions provided the answer. Supervision by itself did not guarantee that participants would read or follow the instructions.

## Data Availability

All data and materials are available upon request.
